# Serum levels of lycopene, beta-carotene, and retinol and their correlation with sperm DNA damage in normospermic and infertile men

**Published:** 2015-12

**Authors:** Taiebeh Ghyasvand, Mohammad Taghi Goodarzi, Iraj Amiri, Jamshid Karimi, Marzieh Ghorbani

**Affiliations:** 1 *Department of Clinical Biochemistry, Hamadan University of Medical Sciences, Hamadan, Iran.*; 2 *Research Center for Molecular Medicine, Hamadan University of Medical Sciences, Hamadan, Iran.*; 3 *Endometer and Endometriosis Centre, Fatemieh Hospital, Hamadan University of Medical Sciences, Hamadan, Iran*.

**Keywords:** *Infertility*, *Lycopene*, *Beta- carotene*, *Retinol*, *DNA damage*, *Lipid peroxidation*, *Malondialdehyde*

## Abstract

**Background:**

Oxidative stress in reproductive system leads to sperm DNA damage and sperm membrane lipid peroxidation and may play an important role in the pathogenesis of male infertility, especially in idiopathic cases. Antioxidants such as carotenoids function against free radical damages.

**Objective:**

The aim of this study was to determine the levels of lycopene, beta-carotene and retinol in serum and their relationship with sperm DNA damage and lipid peroxidation in infertile and normospermic males.

**Materials and Methods:**

Sixty two infertile men and 71 normospermic men participated in this study. Blood and semen samples were collected from all subjects. Sperm DNA damage was measured using TUNEL method. Carotenoids, retinol, and malonedildehyde in serum were also determined.

**Results:**

DNA fragmentation was higher in infertile group comparing to control group. Serum levels of lycopene, beta-carotene and, vitamin A in infertile men were significantly lower than normospermic men (p< 0.001, =0.005, and =0.003 respectively). While serum MDA was not significantly different between two groups, MDA in seminal plasma of infertile men was significantly higher than control group (p< 0.001).

**Conclusion:**

We concluded that lycopene, beta-carotene, and retinol can reduce sperm DNA fragmentation and lipid peroxidation through their antioxidant effect. Therefore the DNA fragmentation assay and determination of antioxidants factors such as lycopene, beta-carotene and retinol, along with sperm analysis can be useful in diagnosis and treatment of men with idiopathic infertility.

## Introduction

Infertility has a high incidence in young couples and more than 50% of the cases are related to the male factors. Approximately 25% of the male infertility causes are idiopathic (1).

Reactive oxygen species (ROS) are released by leukocytes and abnormal sperms (2). Increased ROS production in sperms and reduction in its scavengering capability leads to oxidative stress and can cause infertility in men (3). A set of antioxidants with scavengering properties, maintains a steady state of ROS in seminal plasma. 

Seminal plasma contains a number of antioxidant enzymes, including superoxide dismutase, glutathione peroxidase, glutathione reductase, and catalase, and also non-enzyme antioxidants, such as ascorbate, alpha-tocopherol, urate, coenzyme Q10, albumin, N-acetyl-L-cysteine, pyruvate, taurine and hypurate (3, 4).

ROS attack polyunsaturated fatty acids (PUFA) in sperm membrane and lead to accumulation of lipid peroxidation products and destruction and deterioration of PUFA. This process causes reduction in mobility, fluidity, function and fertility of sperms (5).

Maintaining sperm DNA integrity is important for the transfer of genetic information and fetal development (6). Damage to sperm DNA causes DNA fragmentation, cross-reaction of protein, and low ability of oocyte fertility. ROS can cause genetic mutations including point mutations, and polymorphisms resulting in a decrease in semen quality (7). DNA is modified and changed by mutagens such as ROS, ultraviolet, gamma, X-rays, and also alkali elements (6, 8).

Nearly 90% of dietary carotenoids include alpha- and beta-carotene, beta-krypto-gezantyne, lutein and lycopene having high double bonds show the highest antioxidant activity. Lycopene a member of this group has good antioxidant activity includes singlet oxygen and free radical scavenging (9).

Based on epidemiological studies, a high uptake of dietary carotenoids on tissue is positively correlated with carotenoid concentrations and negatively with high risk of chronic diseases (9-11). The inverse relationship between lycopene and beta-carotene with risk of cardiovascular disease and certain cancers has been identified (9, 12, 13). Unlike the other carotenoids, heat causes the increase of lycopene absorption by converting trans- to cis-isomer; also cis-isomer is more soluble in bile micelles and can be absorbed better (14).

Retinol is a major vitamer of vitamin A that plays a role in the growth and differentiation of the cells. Also it is involved in differentiation of germ cells in the gonads and in spermatogenesis (15). The aim of this study was to determine the levels of lycopene, beta-carotene and retinol in serum and their relationship with sperm DNA damage and lipid peroxidation in infertile and normospermic males.

## Materials and methods

This case control study was performed in Research Center for Molecular Medicine and Endometer and Endometriosis Center, Hamadan University of Medical Sciences, Hamadan, Iran, during 2012-2013. The protocol of this study was approved by the Ethics Committee of Hamadan University of Medical Sciences, and informed consent was obtained from all studied participants.

Two age matched groups (20-40 years) containing 71 normospermic men and 61 men with idiopathic infertility were participated in this study. Infertile men were diagnosed according to world health organization criteria that were referred by an urologist. Infertile men with varicocele, cryptorchidism, renal and urinary tract infections, trauma, chemotherapy, and smokers were excluded from the study. 

Blood and semen samples were collected from all participants 3 to 4 days after the last sexual intercourse using self- arousal. As leukocytes can produce ROS (16), the samples containing white blood cells and immature sperms were excluded from the study.

**Sample preparations and analysis**

The semen analysis, according to World Health Organization protocol (17) was carried out manually and using computer analysis by CASA (Camputer Aided Sperm Analysis) via video test sperm 2.1 software. A serum sample was also prepared from all participants for carotenoid analysis. Prepared serum and semenial plasma samples were maintained at -70^o^C until analysis.

Sperm DNA fragmentation assay was carried out using TUNEL (Terminal deoxynucleotidyl transferas-mediated dUTP nick-end labeling) according to the kit manufacturer protocol (Roche, Cat. No.11684795910, Germany). DNA fragmentation was determined using a fluorescence microscope (Ziess, Germany) at 488 nm. 

Malondialdehyde (MDA) as a lipid peroxidation index was determined both in serum and seminal plasma according to the thiobarbitoric fluorimetric assay (16). The fluorescence intensity was measured at excitation wavelength of 515 nm and emission wavelength of 553 nm (Fluorimete Jasco FP-6200). Lycopen, beta carotene and viamin A were extracted from prepared samples according to the previously reported methods (18, 19).

**Chromatographic conditions**

Chromato-graphic analysis was carried out using Waters HPLC device (isocratic pump, column C18, 150 x 3.9mm, particle size 5µm, Novopack). Mobile phase was consisted of HPLC- grade methanol: acetonitrile: tetrahydrofuran (Merck, 5:50:45 v/v) containing butyl hydroxy toluene 0.01%; flow rate was 0.8 ml/min. Standards for lycopene (9879 Sigma), beta-carotene (Fluka 22040) and retinol (7632 Sigma R) were prepared; also retinyl acetate (Sigma) was used as internal standard. Concentrations of lycopene and beta- carotene and vitamin A in serum were calculated by the application of mellenium 1000 software according to the standard curves.

**Statistical analysis**

Data analysis was performed using Pearson and t-test in Statistical Package for the Social Sciences, version11, SPSS Inc, Chicago, Illinois, USA (SPSS software). p<0.05 was considered significant.

## Results

The mean (SD) age of the participants in normospermic and infertile groups was 32.40 (3.9) and 34.9 (4.5) years respectively, showing no significant difference (p= 0.120). Results of sperm analysis are shown in table I. Comparison of means of morphology, sperm count, mobility, and viability between the two groups showed significantly lower levels in all these parameters in infertile men (Table I).

**Figure 1:**
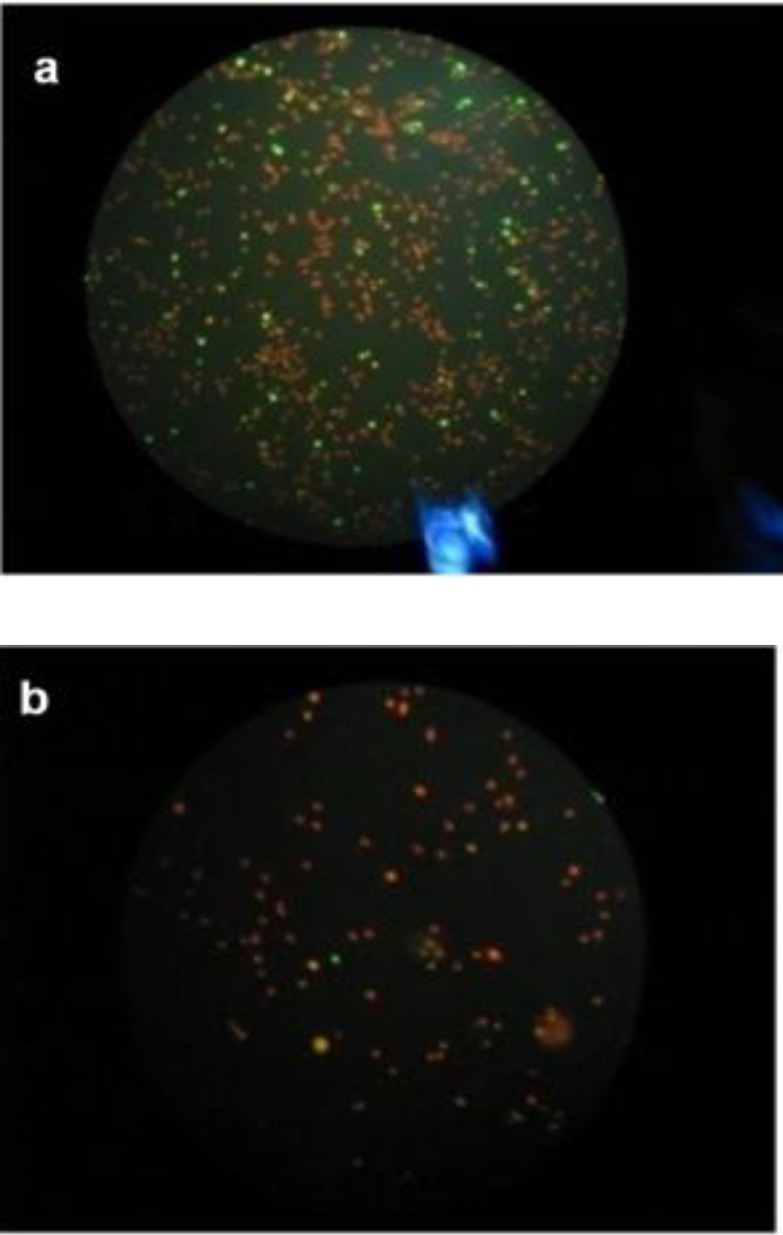
An image from TUNEL assay results. a) Infertile b) control, Damaged sperms are in green and normal sperms in red color.

DNA fragmentation rate in infertile male was higher compared to the control group (p<0.001). Figure 1 shows a typical TUNEL test results, indicating higher number of sperm with fragmented DNA in an infertile men sample. Sperm DNA fragmentation rate was higher in infertile men compare to normospermic men (18.94 ± 2.68 vs. 13.42 ± 2.14 %, p< 0.001).

The results of MDA assay are presented in table I. The findings showed while the level of serum MDA was not significantly different between the two groups; in seminal plasma it was significantly higher in infertile men comparing to control group (p< 0.001).

**Figure 2:**
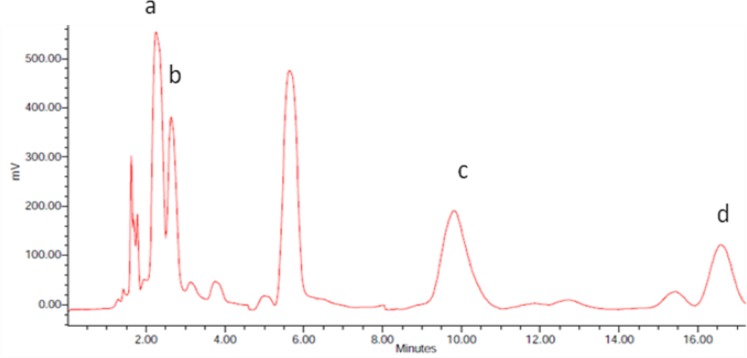
A chromatogram of measured compounds in a serum sample. a) retinol b) retinyl acetate c) lycopen d) beta caroten

Lycopene, beta-carotene, and vitamin A levels in infertile men were significantly lower than those of normospermic men (Table I). Figure 2 shows a typical chromatogram of the HPLC analysis. Relationship between serum lycopene and MDA of seminal plasma with sperm mobility and TUNEL test results were determined in both groups. Also the relationship of vitamin A and beta-carotene with these parameters were calculated. Significant correlation between seminal plasma MDA level and sperm DNA fragmentation was observed (r= 0.3 p= 0.02). The inverse relationship between DNA damage and serum lycopene was significant (r= -0.3 p= 0.048). There was an inverse significant relationship between serum beta- carotene and serum MDA concentrations in normospermic subjects (r= -0.1 p= 0.001).

**Table I:** The results of semen analysis; serum and seminal plasma MDA; and serum lycopen, beta-caroten and vitamin A in normospermic and infertile men

**Test Parameter**	**Normospermic**	**Infertile**	**P-value** *
Volume (ml)	3.2 ± 0.62	2.6 ± 0.57	0.004
Count (10^6^/ml)	75.6±12.94	53.7±28.96	<0.001
Morphology (%)	30.21±4.8	18.62±11.68	<0.001
Viablity (%)	55.98±6.94	31.32±10.98	<0.001
Mobility (%)	37.86±7.35	17.69±8.96	<0.001
Serum MDA (nmol/ml)	3.49±2.71	3.80±1.60	0.426
Seminal Plasma MDA (nmol/ml)	0.69±0.248	0.93±0.34	<0.001
Serum Lycopen (ppm)	0.29±0.15	0.20±0.12	<0.001
Serum beta carotene (ppm)	0.33±0.19	0.25±0.14	0.005
Serum Vitamin A (ppm)	66.46±15.30	58.78±12.73	0.003

All data are expressed as mean ± standared deviation (SD).

* p values are the results of comparing data between normospermic and infertile men using independent t-test.

## Discussion

The results showed all sperm parameters were significantly lower in infertile group comparing to normospermic men. A problem in diagnosis of male infertility is very close overlap in the semen parameters of fertile and infertile men. Many of the men with normal parameters according to standard semen analysis remain infertile and routine semen analysis does not provide complete information for identification. Therefore for accurate differential diagnosis more specific tests are needed.

Seminal plasma MDA was higher in infertile men, indicating higher ROS production or weaker antioxidant defense in these subjects. Prospective studies have indicated that men with high levels of ROS have seven times less chance of fertilizing compared with men with low ROS levels (20). It has been known that ROS results in sperm cell damage and its high values have a negative relationship with sperm number and motility (20). 

Tavilani *et al* measured MDA in sperm and seminal plasma and indicated that lipid peroxidation has a deleterious effect on the semen quality (21). 

Various researchers reported a direct correlation between the amount of lipid peroxidation in sperm and sperm dysfunction (20, 22). Poor penetration of the sperm into the oocyte in oligospermia men with high levels of ROS has been identified (23). 

According to the obtained results from TUNEL assay, the sperm with DNA damage was higher in infertile men as compared to normospermic men. Increased sperm DNA damage in infertile men has also been reported previously (6, 7). Sperm DNA can be a predictor of pregnancy success rate; lack of DNA repair may increase the fetal abnormalities, pregnancy loss and spontaneous abortion (24). Due to the limitations of routine semen analysis, sperm nuclear DNA status was evaluated as a potential biomarker of reproduction. The integrity of genetic testing provides independent information of sperm quality. Several methods are used to evaluate DNA damage such as TUNEL which can identify DNA strands directly with a high diagnostic value. There are reports showing the higher rate of DNA damage by TUNEL in sperm is associated with lower fertility rate (25, 26).

Sergerie *et al* measured sperm DNA fragmentation using TUNEL and concluded that this method is highly valuable indicator of male fertility (27). Also Trisini *et al* studied the association between semen parameters and comet assay results and indicated that comet assay provides additional information on sperm function (28). 

Many studies have demonstrated that low levels of the antioxidant capacity of seminal plasma are associated with male infertility (29, 30). Total antioxidant capacity of seminal plasma in infertile men is lower than that in fertile men (31). Tomato contains high level of lycopen, therefore tomato rich diet can increase serum lycopene level and reduce oxidative stress (32).

Also a research shows consumption of a “standard size portion” of tomato products can increase plasma and milk lycopen in lactating women (33). 

The presence of lycopene in human semen has been shown and it can be increased after dietary supplementation with a natural source of lyopene (34). 

Our results also indicated the lycopene, beta- carotene and vitamin A levels in the serum of infertile men are significantly lower than those in normospermic men. Gupta *et al* investigated the effects of dietary lycopene on 30 men with idiopathic infertility and noted that lycopene therapy improves the reproductivity by promoting spermogram parameters, resulting in an increase in sperm count and mobility and improvement of sperm morphology (35). 

Palini *et al* measured vitamin A, vitamin E and carotenoids including alpha-carotene, lutein and lycopene as antioxidants and MDA as an index for determining the lipid peroxidation in plasma and semen of infertile and fertile men and investigated their relationship with sperm parameters such as concentration, mobility and morphology (36). They concluded seminal MDA correlates negatively with semen motility and morphology, while antioxidant level correlates positively with semen parameters (36). 

To improve interpretation of the results it would be better to measure these factors in seminal plasma. We tried to measure carotenoids in seminal plasma using HPLC but we did not obtain reliable results. Sheena *et al* also tried to measure carotenoids in seminal plasma but it was not successful (37). 

Zini *et al* preincubated washed sperm suspensions with and without lycopene and then incubated with H2O2 and examined the sperm mobility and index of DNA fragmentation (38). Their results showed a significant reduction in sperm mobility and significant increase of sperm DNA defragmentation in lycopne untreated samples while the treated samples had a significant reduction in sperm DNA defragmentation (38). They concluded preincubation of the spermatozoa with lycopene protects it from oxidative DNA damage in vitro (38). Some studies have shown that vitamin A has a positive role in spermatogenesis of rats and is effective in differentiation of germ cells in gonads and spermatogenesis action, because animals with vitamin A deficiency suffer from infertility and their differentiation of germ cells cannot be observed (36, 15). Assessment of relationship between the studied factors showed a direct association between sperm DNA fragmentation and seminal plasma MDA level and also an inverse relationship between serum lycopene levels and MDA. 

## Conclusion

Our results showed that in addition to routine tests in the diagnosis of male infertility, other tests such as TUNEL assay and MDA measurement can be used to determine the health of genetic content and membrane of sperm respectively. The indices of serum carotenoid antioxidants such as lycopene, beta-carotene and vitamin A can be used in order to determine the effectiveness of environment surrounding sperm in protecting from free radicals. If idiopathic infertility is diagnosed and show oxidative stress origin, anti-oxidants such as lycopene can be used to decrease oxidative stress and increase the chances of fertility.
